# Dissecting racial disparities in multiple myeloma

**DOI:** 10.1038/s41408-020-0284-7

**Published:** 2020-02-17

**Authors:** Catherine R. Marinac, Irene M. Ghobrial, Brenda M. Birmann, Jenny Soiffer, Timothy R. Rebbeck

**Affiliations:** 1 Department of Medical Oncology, Dana Farber Cancer Institute, Boston, MA 02215 USA; 2 Department of Epidemiology, Harvard T.H. Chan School of Public Health, Boston, MA 02115 USA; 3 The Center for Prevention of Progression of Blood Cancers, Dana-Farber Cancer Institute, Boston, MA 02215 USA; 4 Harvard Medical School, Boston, MA 02115 USA; 5 Channing Division of Network Medicine, Department of Medicine, Brigham and Women’s Hospital and Harvard Medical School, Boston, MA 02115 USA; 6 University of Miami Miller School of Medicine, Miami, FL 33136 USA

**Keywords:** Cancer epidemiology, Myeloma, Cancer prevention

## Abstract

Multiple myeloma (MM) is a fatal plasma cell dyscrasia with a median overall survival of 5 to 10 years. MM progresses from the more common but often subclinical precursor states of monoclonal gammopathy of undetermined significance (MGUS), and smoldering multiple myeloma (SMM) to overt MM. There are large racial disparities in all stages of the disease. Compared with Whites, Blacks have an increased MGUS and MM risk and higher mortality rate, and have not experienced the same survival gains over time. The roots of this disparity are likely multifactorial in nature. Comparisons of Black and White MGUS and MM patients suggest that differences in risk factors, biology, and clinical characteristics exist by race or ancestry, which may explain some of the observed disparity in MM. However, poor accrual of Black MGUS and MM patients in clinical and epidemiological studies has limited our understanding of this disparity and hindered its elimination. Disparities in MM survival also exist but appear to stem from inferior treatment utilization and access rather than underlying pathogenesis. Innovative and multidisciplinary approaches are urgently needed to enhance our understanding of disparities that exist at each stage of the MM disease continuum and facilitate their elimination.

## Introduction

Multiple myeloma (MM) is a plasma cell dyscrasia characterized by the proliferation of clonal plasma cells in the bone marrow and the production of monoclonal immunoglobulin and/or light chain, with subsequent end-organ damage^1^. It is the second most common hematologic malignancy in the US, with over 30,000 new cases diagnosed annually^2^, and is becoming increasingly more common^3^. Novel therapies have improved the estimated life expectancy of MM patients from a 5-year relative survival rate of 35% in 2000 to over 50% today^4^. Nevertheless, MM remains incurable and fatal, with most patients dying of the disease.

MM is always preceded by monoclonal gammopathy of undetermined significance (MGUS) and smoldering multiple myeloma (SMM)^5,6^. Those clinically detectable but asymptomatic premalignant conditions progress to malignant MM in a subset of patients for reasons that are poorly understood. Our limited understanding of disease progression is due, in part, to the limited study populations on which estimates of prevalence and progression risk are based. MGUS is present in roughly 3% of the general population aged 50 years or older^7^, and progresses to overt MM at a rate of about 1% per year^8^. However, in some patients, the risk of progression is reported to be as high as 58% in 20 years^9^. SMM has an annual risk of progression of 10% in the first 5 years, but in some patients, the risk is as high as 70% in 5 years^10^. MGUS and SMM are often diagnosed incidentally by serum protein electrophoresis (SPEP) ordered for a differential diagnosis of anemia, bone pain, or renal insufficiency. Even when clinically diagnosed with these earlier phases of disease, most patients do not receive treatment until their disease progresses to symptomatic MM, often manifesting as overt end-organ damage.

## Racial disparities in the incidence of MM and its premalignant conditions: a snapshot

Large racial disparities exist in MM and its premalignant conditions. Here, we define White and Black to be the racial self-identification provided by participants in studies, population registries or administrative databases such as the US Census. Compared with Whites, Blacks have a twofold increased risk of MM^11^ and are diagnosed with MM at younger ages^12^. Disparities in the incidence of MM for other races in the US are less dramatic^13,14^; the incidence of MM is markedly lower in Asians compared with non-Hispanic Whites (incidence rate of 3.8 per 100,000 vs. 6.2 per 100,000), whereas Hispanics have a slightly higher incidence rate than Whites (6.7 per 100,000)^14^. Disparities in the premalignant condition of SMM are not well documented due to the lack of International Classification of Diseases (ICD) codes differentiating SMM from MM. The lack of a systematic disease classification makes clinical and epidemiologic investigations difficult. However, Blacks in the US have been shown to have a markedly higher prevalence of MGUS compared with Whites in several different study samples^15,16^. The disparity in the prevalence of MGUS between Blacks and Whites is especially pronounced at younger ages^17^. Among 12,372 people aged 10–49-year-old in the National Health and Nutrition Examination Survey (NHANES) III, MGUS prevalence was fourfold higher in Blacks compared with Whites in individuals of all ages (0.88% in Blacks vs. 0.22% in Whites; *p* = 0.001), and more than six times higher in Blacks among persons aged 40–49 years (3.26% in Blacks vs. 0.53% in Whites; *p* = 0.0013)^17^. Similar to the incidence of MM in Hispanics, Mexican-Americans appear to have a prevalence of MGUS (0.41%) that is intermediate of that of Whites and Blacks^17^. Further, differences in the prevalence of MGUS have also been observed in a population-based sample of Black men in Ghana, who exhibited a twofold increased prevalence of MGUS compared with a White reference population^18^. This result suggests that the disparity is not limited to Blacks in the US.

Given the extreme disparity observed for Blacks compared with Whites, and the limited data that exist for other racial or ethnic groups, we will herein focus on the disparities observed for Blacks. We summarize the disparities across the MGUS and MM disease continuum with a predominant focus on trends that exist in the US and describe factors that may contribute to these disparities. We also discuss potential strategies for eliminating the disparities.

## Possible causes of observed disparity in risk of MGUS and MM

The causes of the racial disparity in MGUS and MM are complex. Factors that might contribute to or exacerbate the excess risk observed in Blacks compared with other racial/ethnic groups are likely similar for both MGUS and MM, given that MGUS and MM share many of the same underlying risk factors. These factors may include socioeconomic factors, genetics or differences in exposure to established MGUS and MM risk factors (e.g., obesity). However, the available evidence is insufficient to establish causality for most of these factors.

### Socioeconomic factors

Socioeconomic status (SES) has been identified as a risk factor for many cancers^19^, potentially through its correlation with lifestyle and environmental risk factors or its effect on the access and utilization of healthcare services. SES varies by race^20^, and thus it is plausible that at least some components of SES contribute to the racial disparity in MM. However, at present, the contribution of SES to the increased risk of MM among Blacks is not clear. A population-based case–control study of 206 Black and 367 White MM cases and 2131 controls found that a low occupation-based SES score, estimated from average earnings and training required for a job, was significantly associated with an increased risk of MM^21^. Notably, the risk of MM associated with low a SES score was slightly higher for Blacks compared with Whites, supporting the notion that SES contributes to the racial disparity in MM risk between Black and Whites beyond its contribution to MM risk itself. However, a second study of racial disparities in the precursor condition of MGUS suggests that the excess disease prevalence among Blacks is not attributed to differences in SES. In that study, Landgren et al.^15^ examined risk factors for MGUS in a sample of White and Black women of similar SES as determined by educational attainment and annual household income. The authors found that Black women had roughly a twofold greater prevalence of MGUS compared with White women (odds ratio: 1.8, *p*-value = 0.04) even after further adjustment for these education- and income-based SES variables. Thus, the disparity in MGUS prevalence appeared independent of SES^15^. These two studies of race and SES differed in endpoint (e.g., MM vs. MGUS) and SES measure utilized. Thus, the relationship of SES with racial disparity observed in MM remains unresolved. Additional studies that examine the influence of SES or other aspects of the social environment are warranted, specifically in large, racially diverse, and prospectively followed populations.

### Family history and genetic susceptibility

Accumulating evidence also suggests that heritable factors may influence the disparity in MGUS and MM risk between Whites and Blacks. Some of the strongest evidence for heritable factors underlying racial disparities in MM has come from studies demonstrating that having a family history of MM or a related plasma cell dyscrasia (such as MGUS) is a strong and consistent risk factor for both MGUS and MM^22,23^. While the data remain limited, clustering of MGUS and MM has also been reported in black families^24,25^, and there is a suggestion that the excess risk of MM attributed to having a positive family history of MM may be greater in Blacks than Whites^26,27^. In a population-based case–control study, Brown et al.^27^ reported an increased risk of MM among those with a family history of the disease, which was greater in Blacks (OR: 17.4, 95% CI: 2.4 to 348) than in Whites (OR: 1.5, 95% CI: 0.3 to 6.4)^27^. This stronger association of MM with family history in Blacks was recently replicated in a case–control study of individuals enrolled in the Molecular And Genetic Epidemiology (iMAGE) study of MM^26^, in which the odds ratio for MM associated with family history was 20.9 (95% CI 2.59 to 168) in Blacks compared with 2.04 in Whites (95% 0.83 to 5.04). Of note, the sample sizes in both studies were relatively small and effect estimates were imprecise. Nonetheless, the hypothesis that a portion of the disparity in MM incidence between Whites and Blacks is due to heritable factors is further supported by preliminary data from a recent meta-analysis of Genome-Wide Association Studies (GWAS), which suggest that some MM risk loci in Blacks are distinct from the risk loci identified in European Americans^28^. It is notable, however, that most GWAS efforts to date in MM have included predominantly European American cases and controls, and thus the current understanding of MM susceptibility in Black and other non-European American populations remains extremely limited.

### Obesity

Obesity has emerged as a consistent and well-established risk factor for MM^29,30^ and has been associated with an increased prevalence of MGUS^15^. Obesity is also more prevalent in Blacks than Whites^31^. For example, data from NHANES suggest that approximately 48% of all non-Hispanic Blacks have a body mass index (BMI) in the obese range, compared with 34.5% among non-Hispanic Whites^32^. Thus, it has been hypothesized that the excess prevalence of obesity among US Blacks may in part explain the observed disparity in MGUS and MM by race. Blacks also have a higher prevalence of obesity-related medical comorbitities^33,34^, including some that are putatively associated with MM risk such as diabetes^35^. These observations further support the plausibility of obesity and related comorbidities explaining part of the difference in MGUS and MM risk between Blacks and Whites. However, the high correlation between race, obesity and obesity-related comorbidities has made the influences of each exposure on MGUS and MM risk difficult to dissect and presents unique challenges for epidemiologic investigations on the topic, particularly for studies of MGUS. For example, MGUS is asymptomatic, and the likelihood of a diagnosis is closely tied to an increased frequency of comorbidities (and doctor visits)^36,37^. Therefore, Black patients may be systematically diagnosed with MGUS at a greater frequency or at younger ages because of elevated medical intervention due to obesity and related comorbidities. Alternatively, they could be systematically underdiagnosed because of inadequate or less adequate medical care^38^. As elimination of this source of bias in studies of individuals with MGUS is difficult, examination of these complex and interrelated exposures in screening populations will be important for clarifying the independent contribution of race to MGUS risk, as well as its progression to MM.

## Is Black race a risk factor for MGUS progression to MM?

An unresolved question is whether the disparities in MM incidence can be attributed fully to the higher prevalence of MGUS in Black populations, or whether differences in rates of MGUS progression to MM between Blacks and Whites also contribute to the disparity. A 2006 study of MGUS patients diagnosed between 1980 and 1996 in the Veterans Health Administration database did not report a statistically significant difference in the risk of progression to MM for Black MGUS patients compared with White patients^16^. A more recent investigation in the same database included MGUS patients diagnosed between 1999–2009 and found Blacks to have a twofold increased risk for the malignant transformation of MGUS to MM after controlling for other measured risk factors, such as obesity^36^. The discrepant findings across the two Veterans Health Administration studies could stem from differences in the sample size and corresponding power to detect statistically significant associations, or from differences in the criteria used for case identification and/or statistical modeling. Thus, the question of disparity in risk of MGUS progression to MM requires further study and replication in other populations.

## MM mortality and survival outcomes by race

Given the dramatically higher incidence of MM among Blacks compared with Whites, it is not surprising that Blacks also have a higher rate of MM mortality^4^. Estimates from recent US Surveillance, Epidemiology, and End Results Program data indicate that the 5-year age-adjusted mortality rate of Blacks is 6.2 per 100,000, compared with 3.1 per 100,000 in Whites^14^. However, comparisons of survival patterns across racial groups of MM patients, a metric that is not dependent on disease incidence, indicate that Blacks experience similar survival to Whites^14^. For example, the 5-year relative survival appears to be relatively comparable at 53.9% and 51.3% for Blacks and Whites in the US, respectively^14^. Moreover, when treatment is standardized, there is some evidence that Blacks have the potential to experience superior survival after MM diagnosis^39^. For example, in an analysis of 174 Black and 279 White MM patients who underwent autologous stem cell transplantation between 2000 and 2013, Bhatnagar et al.^39^ found that Black patients had better overall survival compared with White patients when survival time was measured from the time of diagnosis to death (median survival time 7.7 years in Blacks vs. 6.1 years in Whites; *p* = 0.03). Conversely, several secondary analyses of clinical trials in the US suggest minimal differences in survival among Black and White MM patients enrolled in MM treatment trials^40–42^, although it is notable that the enrollment of Black and other minority patients into MM clinical trials has been extremely low. The small numbers of Blacks enrolled in clinical trials not only limit the confidence in the reported results, but they raise questions as to whether the trends observed in the Black patients who did enroll are generalizable to broader patient populations. Generalizability of results is of particular importance given the elevated rates of MM-related comorbidities (see above) that may lead to exclusion of patients with these comorbidities.

The observation that Black patients with MM may have superior survival compared with White patients^39^ suggests that Blacks have a more indolent disease subtype. This hypothesis is supported by a study from Greenberg et al.^43^, who compared the primary cytogenetic abnormalities in 292 Black and 471 White patients^43^. In that study, Blacks had a lower prevalence of several cytogenetic abnormalities, including *t*(4;14), which characterizes a subtype of MM classified by the International Myeloma Federation as “high risk”^44^. Recent evidence also suggests that Black patients with MM may have a slightly lower prevalence of *TP53* mutations compared with Whites^45^, a mutation that is typically associated with worse prognosis^46^ and therefore consistent with the notion that Black patients have a less aggressive disease subtype. In addition, some but not all analyses have found Blacks to have a higher prevalence of *t*(11;14)^45^, which is an immunoglobulin heavy-chain (IgH) translocation that is associated with standard (non-elevated) prognostic risk^47,48^. For example, a recent study of 68 MM patients (47 White, 21 AA) that used a targeted next generation sequencing assay to characterize genetic alterations found *t*(11;14) was significantly more frequent in Blacks compared with White patents (29% vs. 0%; *p* = 0.001)^45^. While this finding was not replicated in the CoMMpass cohort^45^, findings are consistent with a 2018 study by Baughn et al.^49^, which used genomic ancestry instead of self-reported race to separate population subgroups. In that study, the authors reported that individuals with the highest degree of African ancestry (≥80% African ancestry) displayed a higher prevalence of *t*(11;14), as well as a lower prevalence of the high-risk 17p deletion or monosomy 17, compared with those with the lowest degree of African ancestry (<0.1% African ancestry)^49^.

Despite the data suggesting that Black patients with MM have a more favorable prognosis and may exhibit a better response to several newer therapies than White patients, it appears that the steady increase in survival rates resulting from advancements in MM therapies since the 1990s has been experienced primarily by White patients^12,50,51^. In a population-based study of White and Black MM patients in the SEER-9 database diagnosed between 1973 and 2005, Waxman et al.^12^ found that the magnitude of survival improvement among Blacks after the introduction of novel MM therapies was <50% of the benefit seen in White patients. The authors hypothesized that the observed disparity may be due to socioeconomic factors, such as unequal access to novel therapies and/or differences in treatment response. In a more recent analysis of SEER data with prolonged follow-up, survival improved for all racial/ethnic subgroups except for older and minority patients, including Blacks^51^. This more recent finding is consistent with observations that older, Black and other minority patients have lower uptake rates of autologous stem cell transplantation compared with White patients^52–54^. Emerging data also suggest significant delays in transplant for the minority patients who do eventually receive a transplant after induction therapy, compared with White patients^39,55^. In the study by Bhatnagar et al.^39^ described above, a 1.3-year (standard deviation [SD] = 1.5 years) delay in transplant for Blacks was observed in contrast to a 0.9-year (SD = 1.0) delay for White patients^39^.

Taken together, the evidence suggests that although Black patients have the potential to experience similar or better survival after MM diagnosis compared with White patients, significant disparities in treatment use, access, and referral patterns persist that may impair clinical outcomes. The causes of these treatment-related disparities are not well understood. They may stem from systemic distrust in the medical community^56^, poor accrual of Blacks to clinical research studies^42^, or differences in healthcare access and delivery, geographic location or insurance coverage^57^. Understanding and targeting the causes of these treatment-related disparities in MM is critical to achieve more equitable treatment delivery and outcome for all MM patients.

## Research considerations and future directions for reducing MM racial disparities

### Early detection: benefits and knowledge gaps

The usual clinical management of patients diagnosed with MGUS and SMM is to delay therapy until the patient has progressed to overt MM. However, observational studies have suggested that increased monitoring of MGUS/SMM patients could deliver a timelier diagnosis of overt MM and result in fewer clinical disease manifestations^37,58^. In addition, there is accumulating data from clinical trials, suggesting that early intervention in the precursor setting may alter the natural history of the disease^59,60^, and importantly, may improve survival outcomes^61^. Indeed, a landmark study by Mateos et al.^61^ demonstrated that early treatment with Lenalidomide and Dexamethasone in patients with high-risk SMM can delay progression to overt MM and increase overall survival. These emerging data from clinical trials provide the strongest evidence for a substantial clinical benefit to diagnosing patients earlier in the disease process. This benefit of early detection is likely to increase as trials for MGUS and SMM become more widely available and/or early treatment becomes the standard of care. However, the degree to which such early detection and intervention efforts benefit Blacks is unclear due to low accrual of minority patients in most trials. Therefore, there is a particular need for studies of early interventions in non-Whites.

### Risk stratification

As MM is considered incurable once it develops, targeted disease prevention and early detection are critical. An important and unresolved question is how to identify individuals at the greatest risk for MM, e.g., those who would be the most suitable candidates for specific types of intervention efforts mentioned above. The most effective strategies will likely involve interventions at the MGUS and SMM stages. At present, standard risk stratification tools for identifying “high-risk” patients with precursor lesions^62^ only consider clinical characteristics of the disease, such as M-protein level, heavy-chain type, and free light chain ratio. Other factors that influence progression to MM in patients with precursor conditions are not well known, although evidence summarized in this review suggests roles for non-modifiable (family history/genetic predisposition) and modifiable factors (obesity). Further research is warranted to evaluate the most informative profile of clinical and non-clinical factors to integrate into risk stratification tools.

The consideration of genomic signatures may improve the ability to identify patients at the greatest risk for progression of MGUS and SMM to malignant MM. This knowledge may also be beneficial in developing targeted/personalized interception strategies. Importantly, these signatures may aid in the reduction of disparities if the molecular signature can help to explain differences by race or ancestry and identify optimal intervention modalities. MGUS/SMM clones may already harbor genomic alterations that influence progression to MM^63^. It is unclear if racial subgroups differ in these disease sub-clones in a manner that would facilitate risk stratification, as reports of differences in these molecular events by race or across risk factors have been extremely limited. Evidence supporting the hypothesis that molecular differences in the disease exist by race have primarily come from data collected in large patient cohorts or population-based samples of patients with overt MM. Those studies have observed genomic heterogeneity by race. For example, as mentioned previously, differences in the primary cytogenetic subtypes in MM patients have been reported across cohorts of Black and White MM patients^43,64^. In addition, a recent study using somatic whole exome, RNA-sequencing and clinical data from 718 MM patients from the Multiple Myeloma Research Foundation CoMMpass study identified several genes—all of which had known involvement in translocations in B-cell malignancies—that had significantly higher mutational frequencies in Blacks compared with Whites^65^. We are not aware of comprehensive molecular studies that have examined differences in genomic events by race in the precursor MM disease stages or attempted to characterize their impact on progression to MM. However, differences in the prevalence of immunoglobulin isotypes have been observed across racial/ethnic groups of MGUS patients, which is consistent with the hypothesis that there is a biological basis for disparities arising in precursor lesions. For example, compared with Whites, Black MGUS patients have been shown to have lower rates of immunoglobulin M (IgM) MGUS, a subtype that tends to progress to Waldenström macroglobulinemia, and higher rates of unquantifiable immunoglobulins^18,66,67^. Molecular studies among racially diverse populations of MGUS and SMM patients could greatly enhance the identification of subgroups of MGUS/SMM patients with an elevated risk of disease progression and who might benefit from early intervention. Such studies would also provide valuable insights into the contribution of molecular features to racial disparities across the disease continuum.

### Screening

Given that disparities in MM incidence and mortality may hinge on events occurring early in myelomagenesis, early detection of precursor lesions is critical for the reduction of MM disparities. Screening for MGUS and SMM is not currently performed routinely. Instead, those conditions are most often diagnosed incidentally when a physician orders a SPEP for a differential diagnosis of anemia, bone pain, or renal insufficiency. Screening for early cancer detection has been implemented in many settings, including prostate cancer (with prostate-specific antigen and digital rectal exam), breast cancer (with mammography), and colon cancer (with colonoscopy). These modalities have demonstrated notable successes but also have limitations in terms of under-utilization, especially among Blacks^68^, and over-diagnosis. A simple blood test can accurately detect the presence of a plasma cell dyscrasia, highlighting the feasibility of screening individuals for these precursor conditions.

Despite its feasibility, screening entire populations for MM is not likely to be cost effective. Instead, screening would be more effective when targeted to high-risk populations as defined by risk factors, including individuals who are Black^69^, have a positive family history of MGUS or MM, or are of older age. These groups could be further stratified into groups at increased likelihood of progression to MM by using molecular and other risk factors described above. However, nomograms or algorithms that identify Blacks at particularly elevated MM risk, and who may benefit most from early interventions, currently do not exist. The potential to leverage knowledge of MGUS status and relevant measurable co-factors (both modifiable and non-modifiable) to stratify progression risk and guide clinical care of patients with pre-malignancy also represents a compelling opportunity to develop evidence-based clinical practices that could significantly diminish both the burden and disparity of MM. Therefore, screening studies enriched for Blacks and other higher-risk individuals could provide urgently needed insights to guide the development of strategies for the targeted screening of high-risk populations to facilitate effective clinical surveillance and interventions.

## Summary and future directions

Racial disparities in MGUS, SMM, and MM are complex and represent a significant public health and health equity concern. The majority of studies to date examining racial disparities across the MM disease continuum have used self-identified exposures of race, which may not adequately capture genomic variability between some of the societal-based “Whites” and “Blacks” with multi-ethnic ancestry. Thus, there is a clear need for more studies to examine disparities in MGUS, SMM, and MM using genomic ancestry. There is also a need for studies that define molecular mechanisms of clonal evolution early in the MGUS/SMM disease stages for high-risk populations, including Blacks and/or individuals with a high degree of African ancestry. These novel risk factors, biomarkers of disease progression, and prevention and interception strategies can be incorporated into screening tests and other interventions that could prevent or delay progression from MGUS/SMM to overt MM. If these screening protocols can be optimally defined for Black and African ancestry populations, disparities in MM incidence and mortality may also be reduced or eliminated, particularly if they also facilitate receipt of timely, high-quality clinical care. Concerted efforts to understand and target the cause of the observed differences in treatment utilization and access by race/ancestry will be critical for achieving more equitable treatment delivery and outcome for all MM patients.
